# The European Health Data Space and biobanking in Europe: synergies, tensions and the future governance of data-driven health research

**DOI:** 10.3389/fgene.2026.1932078

**Published:** 2026-09-10

**Authors:** Laura Grech, Nikolai Paul Pace

**Affiliations:** 1 Department of Applied Biomedical Sciences, Faculty of Health Sciences, University of Malta, Msida, Malta; 2 Centre for Molecular Medicine and Biobanking, University of Malta, Msida, Malta; 3 Department of Anatomy, Faculty of Medicine and Surgery, University of Malta, Msida, Malta

**Keywords:** 1+ million genomes, artificial intelligence, BBMRI-ERIC, biobanking, dynamic consent, European Health Data Space, federated analysis, GDPR

## Abstract

The European Health Data Space (EHDS) represents the most ambitious attempt to date to create a common legal, technical and governance framework for the use of health data across the European Union. Its adoption creates a new infrastructure for both primary use, focusing on cross-border access to electronic health records for care, and secondary use, centred on research, innovation, public health, regulatory science and policymaking. In parallel, European biobanking has matured into a core research infrastructure supporting genomic medicine, biomarker discovery, rare disease research and precision public health. The convergence of EHDS-enabled electronic health records, BBMRI-ERIC-associated biobanks, and the 1+ Million Genomes initiative offers a plausible route towards federated, large-scale, molecularly informed health systems in Europe. This article critically examines that opportunities and challenges arising from this convergence. It argues that the EHDS and biobanking are complementary but not automatically interoperable. Biobanks provide depth: biospecimens, molecular assays, longitudinal cohorts and consented research infrastructures. The EHDS provides breadth: population-scale clinical trajectories, regulatory gateways, Health Data Access Bodies and Secure Processing Environments. The 1+ Million Genomes and Genomic Data Infrastructure initiatives provide a genomics-specific federated layer that may connect these ecosystems through standards such as GA4GH, Beacon, Phenopackets and FHIR-Genomics. However, substantial obstacles remain. These include uneven digital maturity across Member States, immature representation of biospecimen and omics metadata in clinical data models, unresolved interactions between EHDS opt-out mechanisms and biobank consent, fragmented national interpretations of the GDPR, sustainability constraints, environmental costs of petabyte-scale computing, and the risk that artificial intelligence trained on biased or incomplete datasets will reproduce health inequities. We argue that EHDS-biobank integration should not be regarded solely as a technical exercise. Rather, it represents a governance challenge involving trust, reciprocity, legal interpretation, standards development, infrastructure investment and public legitimacy. Without coordinated action, the EHDS may create a formally integrated yet substantively unequal data ecosystem. Conversely, with deliberate design and effective governance, it has the potential to provide Europe with a trustworthy, federated and clinically actionable research infrastructure.

## 1 Introduction

Health research in Europe is entering a decisive period of institutional restructuring. The European Health Data Space (EHDS) Regulation establishes a Union-wide framework for the access, exchange and reuse of electronic health data (European Union, 2025). It is designed to address a long-standing European paradox: health systems generate immense volumes of clinically valuable data, but these data remain fragmented across institutions, regions and Member States (Horgan et al., 2022). The result is a fragmented landscape in which patients may struggle to access their health records across borders, researchers face inconsistent procedures for data access, and regulators lack harmonised streams of real-world evidence.

Biobanking has evolved along a parallel trajectory. European biobanks have shifted from local specimen repositories to highly structured research infrastructures that integrate biological samples with clinical, genomic, imaging, lifestyle and longitudinal outcome data. The Biobanking and Biomolecular Resources Research Infrastructure—European Research Infrastructure (BBMRI-ERIC) has provided a pan-European coordination framework (Litton, 2018) while national biobank initiatives have become central to genomic discovery, biomarker validation and precision medicine. Yet biobanking remains heterogeneous (Antoniades et al., 2021; Grech et al., 2025). Differences in sample collection, pre-analytical processing, storage, consent, coding, metadata, governance and access procedures continue to limit cross-border reuse (Riegman et al., 2019; Jansen et al., 2026).

The EHDS and biobanking therefore appear to be natural partners, but their integration is technically, legally and ethically non-trivial. Electronic health records (EHRs) and biobanks differ fundamentally in their purpose, temporality and governance (Beesley et al., 2020). EHRs are primarily generated to support healthcare delivery, reimbursement and administrative processes, whereas biobank data are collected to facilitate research, data/specimen sharing under specific consent frameworks and with long-term stewardship obligations. EHRs are dynamic, continuously updated records of clinical encounters, while biobanks are specimen-linked resources whose scientific value depends not only on clinical phenotype but also on biological provenance, sample handling and molecular assay quality (Michalska-Falkowska et al., 2023; Liang et al., 2025). These differences are not merely operational; they determine what can be linked, how data can be interpreted, and what legal, ethical and scientific issues arise when data is reused.

A further dimension is introduced by the 1+ Million Genomes initiative (1+ MG) and its implementation through the Genomic Data Infrastructure (GDI) (Saunders et al., 2019; Vicente, 2024). These initiatives aim to enable secure federated access to population-scale genomic and phenotypic data across Europe while maintaining national control over sensitive datasets. Their overarching goal is to establish a pan-European genomic ecosystem capable of advancing biomedical research, personalised prevention and treatment, and evidence-based health policy. Its scientific utility lies in creating a virtual, cross-border cohort of sufficient scale and diversity to improve rare disease diagnosis, cancer and infectious disease genomics, pharmacogenomics, variant interpretation, population reference panels, and the validation of genomic medicine across European populations (European Union, 2026).

Such initiatives are strategically important because genomic data is not simply another category of health data. It is potentially identifiable, carries familial implications and is computationally intensive (Erlich and Narayanan, 2014). Consequently, their integration into the EHDS requires standards and governance models that extend beyond conventional EHR interoperability (O’Doherty et al., 2021; Yu et al., 2025).

Against this background, this article provides a critical review of the EHDS-biobanking interface. It explores the regulatory architecture of the EHDS, the structure of European biobanking, the role of 1+ MG/GDI, the technical and semantic requirements for integration, the implications for clinical care and research, and the legal and ethical tensions that remain unresolved. Rather than presenting the EHDS as an inevitable solution, this article argues that its success will depend on deliberate choices regarding governance, interoperability, data stewardship and public trust. The central premise is that integrating the EHDS with European biobanking is not merely a technical undertaking but a strategic governance challenge that will determine whether Europe can realise a trustworthy, equitable and scientifically robust health data ecosystem.

## 2 Methods

This review was conducted and reported in accordance with the Preferred Reporting Items for Systematic Reviews and Meta-Analyses (PRISMA) 2020 statement. Because the review question is cross-disciplinary and interpretive—spanning EU regulatory instruments, biobanking infrastructure, genomic-data standards and data governance, the synthesis is narrative rather than quantitative. No meta-analysis or formal risk-of-bias meta-assessment was applied. The review protocol is summarised below.

### 2.1 Review question

What are the technical, semantic, legal, ethical and governance requirements, opportunities and tensions arising from the integration of the EHDS with European biobanking infrastructures and the 1+ Million Genomes/Genomic Data Infrastructure (1+ MG/GDI)?

### 2.2 Information sources

We searched PubMed/MEDLINE from 1 January 2014 to July 2026. This was supplemented by (i) EUR-Lex for primary EU legal instruments (the General Data Protection Regulation - GDPR, the EHDS Regulation, the AI Act and the NIS2 Directive); (ii) targeted searching of official documentation from BBMRI-ERIC, Global Alliance for Genomics and Health (GA4GH), the 1+ MG initiative and the GDI; and (iii) backward and forward citation searching of included records.

### 2.3 Search strategy

Details of search terms, inclusion and exclusion criteria, selection process, and PRISMA flow diagram are presented in the Supplementary Material.

### 2.4 Data synthesis

Included sources were charted against the review’s analytical framework and synthesised narratively. The framework follows an ordered progression: it begins with the three background infrastructures (the EHDS, European biobanking, and the 1+MG/GDI genomic layer), moves to the technical and semantic requirements that their integration demands, then to the implications for clinical care and research, and finally to the legal, ethical and governance tensions that determine whether integration is realised. Each dimension is examined in turn.

## 3 The regulatory architecture of the EHDS

The EHDS is founded on two complementary pillars: primary use and secondary use of electronic health data (European Union, 2025). The primary-use pillar is directed towards clinical care. It aims to give citizens access to their electronic health data and to allow health professionals to access relevant patient information across Member States, subject to appropriate legal and technical safeguards. Priority data categories include patient summaries, electronic prescriptions and dispensations, laboratory results, imaging reports, discharge reports and other structured clinical data. By facilitating cross-border interoperability, this pillar aims to improve continuity of care, reduce unnecessary duplication of investigations, enhance patient safety and support the free movement of citizens within the Union.

The secondary-use pillar is more directly relevant to research and biobanking (van Drumpt et al., 2025). It establishes Health Data Access Bodies (HDABs) as national or regional authorities responsible for assessing requests and granting access to health data for predefined secondary use purposes (Svingel et al., 2025). These purposes include scientific research, innovation, policy development, regulatory activities, public health, personalised medicine and statistics. At the same time, the Regulation establishes clear boundaries for data reuse by prohibiting applications that may adversely affect individuals, including discriminatory decision-making, advertising, insurance risk stratification and other uses that conflict with the public interest or fundamental rights.

The EHDS introduces several innovations that distinguish it from other European data governance frameworks (Julesz, 2023). First, it creates a structured permit system for secondary use. Second, it requires processing within Secure Processing Environments (SPEs), limiting or prohibiting the download of raw individual-level data. Third, it establishes a European infrastructure, HealthData@EU, for cross-border secondary use. Fourth, it creates a new opt-out right for individuals in relation to secondary use, while leaving some details of scope and implementation to Member States. Fifth, it positions interoperability, metadata catalogues, security, auditability and data quality as regulatory requirements rather than voluntary best practice (Hussein et al., 2025; European Union, 2025).

For biobanking, the implications extend beyond improved access to EHRs. The EHDS has the potential to designate many biobanks, disease registries and research infrastructures as health data holders, fundamentally redefining their role within the European research ecosystem (Quinn et al., 2024). Rather than acting solely as custodians of biological samples and associated datasets, biobanks may become regulated participants within the EHDS governance architecture, responsible for demonstrating compliance with requirements relating to data quality, metadata completeness, interoperability, security and transparent governance. Consequently, participation in the EHDS will require organisational as well as technical adaptation, including investment in data stewardship, harmonisation and regulatory compliance.

The EHDS should be understood as a framework rather than a finished operational system. Its practical effects will depend on national implementation, HDAB capacity, technical standards, and the ability of Member States to build interoperable SPEs. The risk is that formal harmonisation may obscure persistent operational fragmentation (Marelli et al., 2023; Kessissoglou et al., 2024). A common Regulation does not automatically create common infrastructure, common interpretation, or common scientific usability.

These mechanisms are necessary but not sufficient. A SPE enforces where computation happens and what leaves it, but it cannot adjudicate whether a given secondary use is compatible with the consent under which a biospecimen was originally collected, nor reconcile divergent national interpretations of that question. A permit system standardises the application, not the substantive legal basis behind each Member State’s decision. The binding constraints are therefore interpretive and institutional rather than infrastructural: the same SPE, connected to two HDABs applying different views of Article 9 (4) derogations, will yield different research entitlements from identical data.

This is borne out by a rapidly expanding implementation literature. One dimension examines how the Regulation reshapes the citizen’s position, tracing the strengthening of individual rights and the opportunities and obligations it creates for meaningful citizen engagement in the governance and secondary use of health data (Saelaert et al., 2023; Cervera de la Cruz et al., 2026). A second dimension focuses on the operational scaffolding the framework demands: national readiness efforts and integration profiles. This is exemplified by initiatives such as IDERHA and the German Medical Informatics Initiative. These show how existing research infrastructures can be connected to the EHDS and how the primary- and secondary-use pillars might be bridged in practice (Hussein et al., 2024; Declerck et al., 2026; Gruendner et al., 2026). A third dimension cautions that regulatory ambition currently outruns institutional capacity. Assessments of data-quality maturity and of the skills and training needs of the health-data workforce shows that data holders, data users and HDABs are not yet uniformly equipped to meet the Regulation’s requirements (Guardado et al., 2026; Khalifa et al., 2026).

## 4 The structure of European biobanking

Biobanks are organised collections of biological material and associated data, stored and governed for research or clinical purposes. Their value lies in the coupling of biospecimens with metadata. A blood sample, tissue block, DNA aliquot or plasma specimen is scientifically meaningful only when linked to reliable information on donor characteristics, phenotype, disease status, sample handling, assay methods and longitudinal outcomes (Catchpoole, 2017).

European biobanking encompasses a diverse landscape of infrastructures, including population biobanks, disease-specific biobanks, cancer tissue repositories, rare disease collections, birth cohorts, longitudinal epidemiological studies, national genomic initiatives and hospital-linked research collections (Lochmüller et al., 2009; Riegman and van Veen, 2011; Leitsalu et al., 2015; Bycroft et al., 2018). This diversity is scientifically valuable, however, it also produces fragmentation (Aarden, 2023; O’Toole et al., 2024).

Fragmentation occurs at several levels. Operational fragmentation arises from differences in sampling protocols and pre-analytic processing. These variables affect downstream outputs from multi-omic analysis (Srinivasan et al., 2002; Gao et al., 2020; von der Heyde et al., 2024). Informational fragmentation arises from inconsistent metadata fields, local disease coding systems, incomplete phenotyping and variable use of ontologies (Sansone et al., 2019). Governance fragmentation reflects differences in consent models, ethics review processes, access committees, material transfer agreements and policies on commercial use (Gottweis and Lauss, 2012). Technical fragmentation arises from incompatible IT systems and variable adoption of common data models (Rossi et al., 2025).

The pan-European biobank network BBMRI-ERIC has attempted to reduce this fragmentation through infrastructure, standards and coordination (Litton, 2018; Aarden, 2025). MIABIS, the Minimum Information About BIobank data Sharing standard, provides a structured approach to describing biobanks, collections and studies (Norlin et al., 2012; Merino-Martinez et al., 2016). BBMRI-ERIC’s Directory, Locator and Negotiator tools support discovery and access (Holub et al., 2016). These efforts are necessary but insufficient for full EHDS integration. The EHDS requires machine-actionable interoperability across clinical, molecular and governance data. Much of European biobanking remains only partially prepared for that requirement.

The central conceptual challenge is that biobanks should not be regarded simply as databases containing associated biological samples. They are long-term stewardship infrastructures. Their legitimacy depends on participant trust, governance integrity, sample quality and long-term sustainability. Any EHDS integration strategy that treats biobanks as ordinary data providers will underestimate the complexity of specimen-linked research. Empirical work reinforces both points. Studies quantifying the actual use of biological samples across national biobanks and hospital collections show how substantial and unevenly documented this resource already is (Tupasela et al., 2025). The assembly of large, harmonised European cohorts also demonstrates the intensive curation and metadata standardisation that rendering such collections interoperable, and therefore EHDS-ready, will demand (Holub et al., 2026).

## 5 The 1+ million genomes initiative and the genomic data infrastructure

The 1+ MG initiative and the GDI are strategically central to EHDS-biobank integration. The 1+ MG initiative was launched to enable secure access to at least one million sequenced genomes and associated clinical data across Europe (Saunders et al., 2019). The GDI operationalises this ambition through a federated infrastructure that allows genomic data to remain under national control while becoming discoverable and analysable through common standards (Pascucci et al., 2024).

This layer is crucial because genomics cannot be managed as a conventional EHR data type. Whole-genome sequencing produces large, complex files; variant interpretation depends on reference genome build, annotation pipeline, population frequency data, phenotype ontologies and clinical context. Genomic data is also inherently identifying and familial (Bernier et al., 2021; Thomas et al., 2024). A person’s genome carries information about relatives and populations, not only about the individual donor (Mintoff et al., 2024; Phillips et al., 2025). This creates risks that cannot be fully addressed by conventional pseudonymisation (Erlich and Narayanan, 2014; Park et al., 2024).

The EHDS can provide the legal and procedural gateway for secondary use of health data, including clinical phenotypes and outcomes. Biobanks can provide consented samples, molecular assays and curated research cohorts. The GDI can provide genomics-specific federation, discovery and analysis capabilities. Together, these infrastructures could enable a researcher to identify relevant biospecimen collections, access harmonised clinical trajectories through a HDAB permit, and analyse genomic data through secure national nodes without moving raw sequence data across borders (Royo et al., 2025).

## 6 Why EHDS-biobank convergence matters

The rationale for integrating the EHDS with European biobanks is strong. Biobanks provide molecular and biological depth through high-quality biospecimens linked to multi-omic data. The EHDS provides longitudinal clinical breadth through EHRs, healthcare utilisation data and population-level health information. The integration of both holds potential to transform European biomedical research.

For genetic association studies, linkage to harmonised EHR data would allow deeper phenotyping, larger sample sizes and cross-border replication. For rare diseases, federated access to biobank-linked clinical records could increase statistical power and reduce diagnostic delay (Saunders et al., 2019). For pharmacogenomics, prescribing and adverse-event data could be linked to germline variants to identify predictors of efficacy and toxicity. For oncology, tumour biobanks could be integrated with treatment pathways, imaging, survival and molecular profiling. For public health, biobanks could support molecular epidemiology, pandemic preparedness and exposome-informed risk modelling.

The clinical implications are equally substantial. A well-governed EHDS-biobank ecosystem could support risk stratification, early diagnosis, pharmacogenetic prescribing, population screening and learning health systems. It could also enable the evaluation of polygenic risk scores and AI-based decision tools across diverse European populations rather than within a small number of dominant datasets (Ding et al., 2023; Moreno-Grau et al., 2024). This is particularly important because tools trained in one ancestry group, health system or socioeconomic context often perform less well elsewhere (Krieger, 2026; Wang et al., 2026).

**FIGURE 1:**
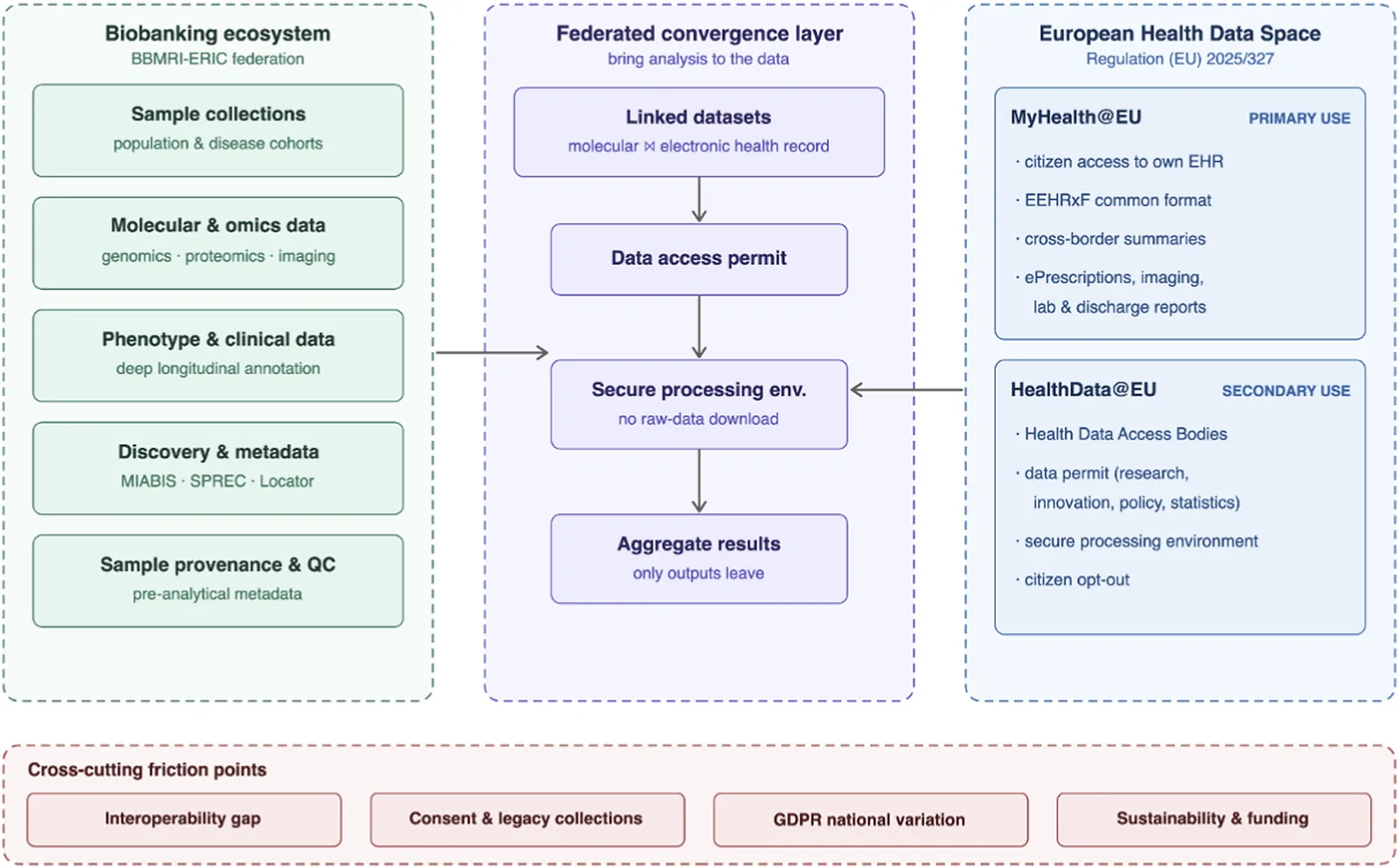
Convergence architecture of the European Health Data Space and European biobanking. The figure summarises the proposed convergence model between European biobanking infrastructures and the European Health Data Space (EHDS). The biobanking ecosystem, coordinated through BBMRI-ERIC and related national infrastructures, contributes specimen-linked resources including population and disease cohorts, molecular and omics data, deeply annotated phenotypic and clinical data, discovery metadata, and sample provenance or quality-control information. These assets are linked to electronic health record data through a federated convergence layer based on the EHDS principle of bringing analysis to the data. Access is mediated through data access permits and implemented within Secure Processing Environments, where raw individual-level data remain protected and only approved aggregate outputs leave the environment. The EHDS component distinguishes MyHealth@EU, which supports primary use for citizen access and cross-border care, from HealthData@EU, which supports secondary use through Health Data Access Bodies, data permits, secure processing, and citizen opt-out mechanisms. The lower panel highlights cross-cutting constraints that may limit implementation, including interoperability gaps, consent and legacy-collection issues, national variation in GDPR interpretation, and sustainability of funding. The figure should be read as a conceptual architecture rather than a complete technical specification; genomic federation through 1+ MG/GDI may be added as an additional omics-specific input layer.

However, the promise of convergence should not be overstated. Linkage does not automatically produce valid inference. EHR data are noisy, incomplete and shaped by care-seeking behaviour, coding practices and reimbursement systems that differ across borders (Kim et al., 2024; Makhni et al., 2025). Biobank participants are often healthier, more educated or more engaged than the general population (Schoeler et al., 2023). For example, a healthy-volunteer bias is well documented in the UK Biobank. Although approximately nine million people were invited to participate, only around 500,000 individuals were enrolled, resulting in a cohort enriched for participants with healthier lifestyles, higher educational attainment, and better overall health than the general UK population (Stamatakis et al., 2021; Schoeler et al., 2023). Biospecimen-derived molecular measurements are susceptible to batch effects and pre-analytical artefacts (Ellervik and Vaught, 2015; Thachil et al., 2024). Federated analysis can reduce data movement but does not remove confounding, bias or poor metadata. The scientific value of EHDS-biobank integration will therefore depend on quality assurance, analytical transparency and explicit modelling of ascertainment bias. The EHDS-biobank interface can be conceptualised as a three-part architecture in which biobank-derived molecular and phenotypic depth is linked to EHDS-enabled clinical breadth through a federated access and analysis layer (Figure 1).

## 7 Technical and semantic requirements for integration

Realising the convergence described above depends on a stack of technical and semantic capabilities. This section examines two that are decisive for biobank-EHDS integration: the federated processing infrastructure on which secondary use depends, and the interoperability required for data to be not merely exchanged but scientifically usable.

### 7.1 Federation, secure processing environments and uneven readiness

The EHDS assumes that Member States can provide secure, auditable and interoperable environments for data processing. This assumption is only partly valid. Digital maturity varies substantially across Europe (Kessissoglou et al., 2024). Some countries have national personal identifiers, mature EHR infrastructures, centralised registries and trusted research environments. Others have fragmented hospital systems, limited structured data capture, weak metadata catalogues and underdeveloped research computing capacity.

The SPE is the technical heart of the EHDS secondary-use model. It implements the principle that analysis should come to the data rather than data being exported to the analyst (Raab et al., 2023). This is particularly appropriate for genomics, where raw data movement is both risky and inefficient. SPEs can enforce authentication, access control, logging, output checking, containerised workflows and restrictions on data export (Rodríguez-Mejías et al., 2024).

However, not all SPEs are equivalent. An environment adequate for tabular EHR analysis may be inadequate for genomics, histopathology or imaging. Omics and imaging analysis requires high-performance storage, specialised software, and GPU acceleration for some tasks. A minimal EHDS SPE could therefore satisfy legal requirements while being scientifically underpowered.

The likely result is a two-tier system unless deliberate investment occurs. Well-resourced Member States and large academic centres will operate advanced SPEs capable of multi-omics analysis. Smaller Member States, regional hospitals and disease-focused biobanks may become nominally connected but practically marginal. This would reproduce the inequalities the EHDS aims to overcome.

A practical strategy could include regional shared SPE hubs, common containerised workflows, centrally maintained reference pipelines, capacity-building for biobank informatics, and targeted funding for underrepresented regions. The infrastructure should be designed not only for compliance but for scientific reproducibility. Every analysis should be traceable to data version, code version, ontology version, consent status and output disclosure controls.

These measures are necessary rather than sufficient conditions. A well-provisioned SPE determines the security and reproducibility of analysis, but it cannot by itself resolve who is entitled to run that analysis, under which national interpretation of the legal basis, or with what recognition across borders.

Recent initiatives showcase both the feasibility and the limits of this model. Working federated infrastructures such as privacy-preserving health-data-space nodes for cross-site linkage (Baumgartner et al., 2024), the EUCAIM cancer-imaging federation (Martí-Bonmatí et al., 2025), and multimodal data-modelling frameworks (Cremonesi et al., 2023) show that analysis can be brought to the data at scale. Yet analyses of the emerging architecture of public genomic-data infrastructures, and of the legal dimensions of anonymisation, accountability and access, make clear that the unresolved frictions are legal and semantic rather than computational (Kogut-Czarkowska and Shabani, 2026; Tangaro et al., 2026).

### 7.2 Interoperability: from data exchange to scientific usability

Interoperability is often treated as a technical problem of data format. This is too narrow. For EHDS-biobank integration, interoperability has at least five layers: syntactic, semantic, sample-level, governance-level and analytical.

Syntactic interoperability concerns the ability to exchange data using agreed formats. Fast Healthcare Interoperability Resources (FHIR) is central here (Bossenko et al., 2024; Phuyal et al., 2026a). Semantic interoperability concerns the meaning of exchanged data. A diagnosis code, laboratory value or phenotype must mean the same thing across sites. This requires controlled vocabularies and robust mappings to Systematized Nomenclature of Medicine Clinical Terms (SNOMED CT), International Classification of Diseases (ICD), (Logical Observation Identifiers Names and Codes (LOINC), Orphanet, Human Phenotype Ontology (HPO) and other ontologies (Gargano et al., 2024).

Sample-level interoperability is more specific to biobanking. It concerns the ability to interpret biospecimens and molecular data in context. Pre-analytical variables such as time to processing, storage temperature, anticoagulant, fixation, tissue ischaemia time and freeze-thaw cycles can determine whether molecular measurements are valid. These variables must be machine-readable and linked to downstream omics files. MIABIS and Standardised Preanalytical Code (SPREC) provide partial solutions, but their integration into EHDS technical specifications remains incomplete (Merino-Martinez et al., 2016).

Governance-level interoperability concerns consent, access conditions, return-of-results policies, commercial-use restrictions and data-sharing permissions. A dataset may be technically linkable but legally unusable if consent or governance metadata are missing. Analytical interoperability concerns whether federated results can be validly combined. Differences in sequencing platform, variant-calling pipeline, phenotype definitions and missing data patterns can generate artefactual heterogeneity.

The practical challenge is therefore not simply to map biobank data to FHIR or Observational Medical Outcomes Partnership (OMOP). It is to build biobank-grade interoperability. This requires implementation guides for biospecimen data, OMOP extensions for genomics and molecular assays, GA4GH integration, persistent identifiers for samples and datasets, machine-actionable consent metadata, adherence to the FAIR (Findable, Accessible, Interoperable, Reusable) principles, and quality flags that allow analysts to assess fitness for use (Wilkinson et al., 2016; Rehm et al., 2021).

A central risk is a form of ‘interoperability in name only’, in which institutions claim compliance based on high-level metadata or nominal adoption of standards, while the underlying datasets remain too heterogeneous, incomplete or poorly annotated to support valid scientific integration. The EHDS should resist this. Dataset catalogues must include quality metrics, provenance, coding completeness, temporal coverage, assay methods, consent limitations and known biases. Discoverability without usability will create frustration rather than research acceleration.

Semantic-interoperability literature maps both the practical routes and the persistent gaps. Systematic reviews and ontology-based consolidation set out how heterogeneous records can be reconciled (Frid et al., 2023; Ambalavanan et al., 2026). Work on FHIR-OMOP transformation shows how clinical-exchange and analytic models can be bridged (Ardel et al., 2026; Witte et al., 2026); and national metadata-catalogue implementations demonstrate how datasets are made discoverable in practice (Palojoki et al., 2024; Gyrard et al., 2025). These collectively illustrate how much mapping, curation and quality annotation genuine usability still requires.

### 7.3 A multi-standard stack for clinical, genomic and biospecimen data

The standards required for EHDS-biobank-GDI integration extend well beyond FHIR alone. FHIR is a healthcare data exchange standard developed by Health Level Seven International (HL7) to support the structured, modular and machine-readable transfer of clinical information between electronic health record systems (Vorisek et al., 2022). It is particularly useful for exchanging patient summaries, laboratory results, prescriptions, imaging reports, diagnoses, procedures and other clinical data across institutions and health systems. The OMOP Common Data Model (OMOP CDM), by contrast, is not primarily an exchange standard but a research-oriented data model that transforms heterogeneous routine health data into a common relational structure for observational analytics, epidemiology, real-world evidence generation and reproducible cohort studies (Fruchart et al., 2024). Together, FHIR and OMOP CDM provide an important clinical interoperability backbone for the EHDS, especially for cohort identification, medication exposure analysis, laboratory harmonisation, outcome ascertainment and cross-site observational research.

However, FHIR and OMOP CDM were not originally designed to capture the full complexity of genomic, biospecimen-linked and multi-omics data. Integration with biobanks and the GDI therefore requires additional genomics-specific standards developed by GA4GH. These include a) Beacon, which enables privacy-preserving discovery of whether a genomic variant is present in a dataset (Rambla et al., 2022) b) htsget, which supports secure access to high-throughput sequencing data without requiring bulk transfer of raw sequence files (Kelleher et al., 2019); c) Phenopackets, which provide a structured computable format for linking phenotypic abnormalities, diseases, biosamples and genomic findings (Jacobsen et al., 2022); and the Variation Representation Specification, which enables consistent and unambiguous description of genomic variants across systems, reference builds and annotation pipelines (Rehm et al., 2021; Wagner et al., 2021).

Disease and phenotype harmonisation also requires controlled vocabularies and ontologies that operate across clinical, laboratory, genomic and rare-disease domains. The Human Phenotype Ontology (HPO) provides standardised terms for phenotypic abnormalities and is particularly important for rare disease genomics and genotype-phenotype correlation. SNOMED CT provides broad clinical terminology for diagnoses, procedures, findings and body structures (Snomed CT International Edition, 2026). LOINC, the Logical Observation Identifiers Names and Codes system, supports the harmonisation of laboratory tests, clinical measurements and diagnostic observations (Yeh et al., 2021). ICD, the International Classification of Diseases, remains central for diagnostic coding, epidemiology, reimbursement and health-system reporting. Orphanet nomenclature provides rare-disease identifiers and classifications that are essential for cross-border rare disease research and clinical interpretation (Gargano et al., 2024).

A credible EHDS-biobank-GDI architecture should therefore adopt a multi-standard interoperability framework, recognising that no single data model can adequately represent the full complexity of clinical, molecular and genomic data. FHIR can support structured clinical data exchange, OMOP CDM can support observational research and real-world evidence generation, GA4GH standards can support genomic discovery and secure sequence access, and phenotype and disease ontologies can preserve biological and clinical meaning across datasets. The objective should not be to force all data into a single common data model. Rather, it should create a coordinated standards ecosystem in which clinical records, biospecimens, molecular assays, genomic variants, phenotypes and governance constraints can be linked with preserved meaning, provenance and analytical reproducibility.

Adopting these standards resolves syntactic and, in part, semantic interoperability, but it leaves the harder problems untouched. Standards specify how a variant or phenotype is represented, not whether a biobank is permitted to expose it, to whom, under which consent, or with what accountability if a finding is clinically actionable. The residual challenge is no longer building platforms or agreeing formats but aligning heterogeneous regulatory interpretations, metadata models and trust frameworks across jurisdictions (Tangaro et al., 2026). This is an alignment problem that no technical specification can, by itself, solve. Work on citizen and professional engagement with personal genomes, and on common interface requirements for genomic data management, points to the same conclusion (Tommel et al., 2023; Resendez et al., 2026).

The risk is institutional misalignment. EHDS governance is built around HDABs and regulatory permits. Biobanking governance is built around consent, access committees and custodianship. GDI governance is built around federated national genomic nodes. If these frameworks are not coordinated, researchers may face sequential approval layers, inconsistent interpretations of consent, divergent security requirements and incompatible metadata catalogues (Forster et al., 2025). The solution is not to collapse these infrastructures into one authority, but to define mutual recognition, common metadata requirements and shared accountability.

## 8 Implications for clinical care and research

The preceding requirements matter because of what they enable, and constrain, downstream. This section turns to the implications of EHDS-biobank integration for clinical care and for research practice.

### 8.1 Clinical translation and the return path to care

The EHDS-biobank-GDI ecosystem could reshape clinical care if implementation is rigorous. The most immediate benefit of the primary-use pillar is continuity of care. However, the deeper clinical transformation lies in the secondary-use feedback loop.

In pharmacogenomics, biobank-derived germline data linked to prescribing and adverse event records could support evidence generation for genotype-guided prescribing (Panconesi et al., 2026; Stocker and Polasek, 2025). In rare disease, federated phenotype-genotype matching could shorten diagnostic odysseys and support variant reinterpretation as knowledge evolves (Chen et al., 2019; Ta et al., 2025). In oncology, tumour biobanks linked to treatment and outcome data could accelerate molecular stratification and real-world evaluation of targeted therapies. In cardiometabolic disease, integration of polygenic scores, biomarkers, medication exposure and outcomes could improve prevention models, provided they are calibrated locally.

The return path from research to care is not automatic. Many biobanks were established for research and lack clinical validation workflows. EHR systems may not be ready to incorporate genomic decision support. Clinicians may lack training in genomics, AI interpretation and risk communication (Schaibley et al., 2022). Patients may not want all results returned, especially where penetrance is uncertain or interventions are limited (Staunton et al., 2024). The EHDS must therefore be connected to clinical implementation pathways, professional education and evidence-based guidelines.

A learning health system requires more than data availability. It requires governance that determines which findings are clinically actionable, how they are validated, who communicates them, how they are stored in the EHR, and how subsequent decisions are audited. Without this, EHDS-biobank integration may produce research outputs that fail to enter care or, worse, premature implementation of poorly validated tools.

These requirements are given concrete shape by studies of the translation pathway itself: appraisals of the European Patient Summary as a vehicle for research (Laleci Erturkmen et al., 2024), analyses of the opportunities and barriers to secondary use in routine care (Siderius et al., 2023; Metsallik et al., 2024), and work on multimodal data modelling to support clinical decision-making (Muraca et al., 2026) together outline what is needed for research findings to re-enter care safely.

### 8.2 Artificial intelligence, fairness and accountability

AI is both a beneficiary of EHDS-biobank integration and a source of risk. Large, multimodal datasets combining EHRs, imaging, pathology, genomics and biospecimens are precisely what modern predictive models require. However, AI systems are only as reliable as the data and assumptions on which they are trained. European health data are not uniformly representative. Biobanks may overrepresent healthier volunteers, majority populations, urban centres or individuals with higher socioeconomic status (Grech and Pace, 2026). EHRs encode health system access, clinical practice, coding incentives and diagnostic bias. Genomic datasets remain skewed towards populations of European ancestry, but even within Europe there is substantial underrepresentation of smaller, isolated, admixed or founder populations (Dajani et al., 2022; Fatumo et al., 2022).

The AI Act will impose obligations for high-risk AI systems, including risk management, data governance, documentation, transparency, human oversight, accuracy, robustness and cybersecurity. For EHDS-biobank research, this means that dataset provenance, bias assessment and performance stratification cannot be afterthoughts. Models should be evaluated across Member States, ancestry groups, sex, age, socioeconomic strata and clinical settings where feasible. Performance drift should be monitored when models are deployed into care.

A critical issue is that federated learning does not automatically solve bias. It allows models to train across distributed datasets without centralising raw data, but it can still amplify biases if participating nodes are unbalanced or if minority populations contribute insufficient signal (Rieke et al., 2020). Federated AI requires careful governance of node weighting, data quality, fairness metrics, validation cohorts and output interpretability.

The EHDS should therefore require dataset diversity reporting for secondary-use projects involving AI. Biobanks should expose metadata on cohort composition, ascertainment, sequencing platform, missingness and phenotyping depth. HDABs should require applicants to state how bias will be assessed and mitigated. For clinical AI, external validation in independent European populations should be treated as a minimum standard.

This is reinforced from two directions. Efforts to consolidate the AI requirements of predictive, preventive and personalised medicine set out what integrated data could enable (Khalifa and Hussein, 2026). Analyses of the barriers to translating AI into routine clinical and laboratory practice shows how far validation, governance and workflow integration still lag behind that promise (Neale et al., 2026).

## 9 Legal and ethical tensions

The technical and clinical opportunities are bounded throughout by a set of legal and ethical tensions. This section considers consent and participant governance, legal fragmentation and regulatory complexity, economic and environmental sustainability, and the broader challenge of converting compliance into legitimacy.

### 9.1 Consent, trust and participant governance

Consent is one of the most complex points of interaction between the EHDS and biobanking. Traditional biobanking has often relied on broad consent, allowing future research use under ethical oversight (Mikkelsen et al., 2019). Broad consent is practical and has enabled long-term cohort studies, but it is increasingly challenged by expectations of transparency, participant engagement and granular control, and empirical work shows that participant attitudes are themselves heterogeneous (Garrison et al., 2016).

Dynamic consent offers a more interactive model (Kaye et al., 2015). Participants can review projects, alter preferences, receive updates and potentially opt into or out of specific categories of use. Meta-consent goes further by allowing individuals to specify how they wish to be asked in different contexts (Ploug and Holm, 2016; Budin-Ljøsne et al., 2017). These models are attractive for new collections, particularly where genomic data, commercial partnerships and AI development are involved.

However, dynamic consent is not a universal solution. It depends on digital access, literacy, identity infrastructure and sustained communication. It may exclude elderly, socioeconomically disadvantaged or digitally marginalised participants. It also risks transforming research participation into a continuous administrative burden. For large legacy biobanks, reconsenting all participants may be impossible because donors may be deceased, untraceable or recruited under older frameworks.

The EHDS introduces an opt-out mechanism for secondary use. Its interaction with biobank consent requires careful clarification. A participant may have given broad consent for biobank research but later exercises an EHDS opt-out. Conversely, a participant may not opt out of EHDS secondary use but may have biobank consent restrictions that prohibit certain uses, such as commercial research or international transfer. HDAB permits must therefore be able to interpret and enforce consent metadata rather than assuming that regulatory permission overrides research consent.

Trust is broader than consent. Biobank participants want assurance that their contributions are used responsibly, that commercial access is transparent, that risks are minimised, and that benefits return to patients and publics (Staunton et al., 2024). Trust also requires accountability. This includes participant representation in governance bodies, public reporting of approved projects, audit trails, sanctions for misuse, meaningful benefit-sharing and clear policies on return of results (Thorogood et al., 2019; Lowery et al., 2024).

The return of individual findings is especially important in genomics (Vears et al., 2023). If a secondary-use analysis identifies a clinically actionable variant, responsibility becomes ambiguous. The researcher may not have a clinical relationship with the participant. The biobank may not have a recontact pathway. The HDAB may issue permits but not provide clinical interpretation. The healthcare system may be able to act but may not have validated the result. Europe needs clearer frameworks distinguishing research findings, analytically confirmed results, clinically validated findings and obligations to recontact.

Technologies of consent are, once again, enabling rather than decisive. A dynamic-consent interface can record and propagate a participant’s preferences, but it cannot by itself reconcile those preferences with divergent national rules on secondary use. It cannot resolve the institutional question of who bears responsibility when a permitted analysis yields a clinically actionable finding. The unresolved problems are governance issues that the interface exposes rather than settles.

The literature documents both the promise and the limits of these instruments. Studies of public attitudes and willingness to grant broad or altruistic consent reveal conditional, context-dependent support rather than blanket endorsement (Braunack-Mayer et al., 2021; Cumyn et al., 2021; Phuyal et al., 2026b). Work on dynamic and meta-consent and on formal trust indicators examines how preferences can be elicited, honoured and monitored over time (Christofidou et al., 2025; Gille et al., 2025; Richter et al., 2026).

### 9.2 Legal fragmentation and regulatory complexity

The EHDS operates within the GDPR but does not erase national variation (European Union, 2016). GDPR Article 9 permits processing of health and genetic data under specific conditions, including scientific research with safeguards, but Member States may impose additional conditions on genetic, biometric and health data. This has produced substantial variation in consent requirements, ethics review, research exemptions, data linkage, genomic data processing and international transfer (Marelli et al., 2023; Staunton et al., 2024).

The EHDS seeks to harmonise access procedures, but it does not fully harmonise all underlying legal bases. HDABs may develop divergent interpretations of scientific validity, public interest, data minimisation, commercial use and opt-out exemptions (Quinn et al., 2024). Data protection authorities may also interpret GDPR interactions differently. Biobanks operating across borders may therefore still face complex multi-layered compliance.

Several legal issues require particular attention. First, the relationship between HDAB authorisation and research ethics approval must be clarified. Duplication would create delay and uncertainty. Mutual recognition may be possible, but only if responsibilities are clearly allocated. Second, the legal status of legacy consent needs harmonised guidance. Third, the treatment of genomic data requires careful alignment between EHDS provisions, GDPR Article 9 (4), national genomic laws and international data-sharing requirements. Fourth, AI development introduces additional obligations under the AI Act, especially where tools are intended for clinical use (European Union, 2024). Fifth, cybersecurity obligations under NIS2 and sectoral health regulations must be integrated into SPE governance.

International transfer remains a critical limitation, since much biomedical research is global. European biobanks collaborate with partners in the United Kingdom, United States, Asia and international consortia. EHDS access does not automatically permit onward transfer. Even when data remain in Europe, non-EU collaborators may require remote access, raising questions about jurisdiction, safeguards and enforceability. For genomics, where re-identification risk is high, this issue is acute.

The cumulative effect is significant. A multi-country project linking cancer biobanks, EHR outcomes, genomic data and AI development may require HDAB permits, ethics approvals, biobank access committee approval, consent compatibility review, data protection impact assessments, SPE accreditation, commercial-use review, AI Act planning and international-transfer assessment (Forster et al., 2025). Each step may be defensible. Together, they may make high-value research slow, expensive and fragile. The policy challenge is to reduce unnecessary duplication without weakening protection.

It would be a mistake to read this variation solely as a defect to be engineered out. Health-system organisation is a Member-State competence under Article 168 of the Treaty on the Functioning of the European Union, and the GDPR deliberately preserves national discretion over the processing of genetic and health data through the Article 9 (4) derogation. The resulting heterogeneity is, in part, a constitutional feature of the Union rather than a mere implementation failure. It reflects the principle of subsidiarity, under which decisions are taken at the most appropriate level and legitimate national differences in the balance struck between privacy, solidarity and research are protected. This has a direct consequence for governance design. Proposals that presuppose *de facto* harmonisation - a single interpretation of secondary use, one consent standard, a uniform ethics-review outcome are thus likely to founder for the same reason the underlying fragmentation exists, as the experience of both the GDPR’s uneven transposition and BBMRI-ERIC’s federated model illustrates (Marelli et al., 2023; Staunton et al., 2024; Tangaro et al., 2026). The realistic objective is therefore not centralisation but interoperability of governance: mutual recognition of permits and ethics approvals, machine-readable expression of nationally-set conditions (for example, through GA4GH Passports and the Data Use Ontology), and coordination mechanisms that let national bodies retain competence while agreeing on interfaces (Alvarellos et al., 2023).

Comparative legal scholarship documents precisely the variation any workable design must accommodate. This includes divergence in national GDPR implementation and in the rules governing cross-border processing of genetic and health data (Molnár-Gábor et al., 2022a; Molnár-Gábor et al., 2022b; Vukovic et al., 2022; Lalova-Spinks et al., 2023), and persistent post-harmonisation fragmentation in research-ethics and secondary-use requirements across Member States (Nastasa et al., 2025; Lukaševičienė and Gefenas, 2026).

### 9.3 Economic and environmental sustainability

Sustainability is frequently underdeveloped in EHDS discussions. SPEs, genomic storage, AI workloads, metadata curation and cybersecurity are expensive. Many biobanks operate on project-based funding that supports sample collection or specific studies but not long-term data stewardship. EHDS compliance may impose new obligations without providing stable financing.

For genomic biobanks, storage and compute costs are particularly substantial. Raw sequencing files, aligned reads, variant call files, multi-omics matrices and imaging data create petabyte-scale requirements. These cannot be handled by underfunded local servers or improvised research IT infrastructure.

A fair EHDS strategy must therefore include financial redistribution. If only wealthy institutions can meet compliance and interoperability requirements, the EHDS will preferentially integrate already powerful centres. Smaller biobanks, rare disease collections and underrepresented populations may be excluded. This would damage both equity and scientific validity.

Environmental sustainability is also material. Large-scale genomic and AI computing consumes energy and water (Bogmans et al., 2026). The climate footprint of digital health infrastructure should be measured and reduced. SPEs should report energy use, power usage effectiveness, renewable energy sourcing and data retention policies. Not all intermediate files need indefinite storage. Workflow design should include compression, tiered storage, deletion policies, efficient algorithms and green procurement. A European health data infrastructure that ignores environmental costs would be misaligned with broader EU climate commitments.

Studies of the sustainability of secondary use, of health-system resilience and of the health-data funding landscape underline that durable infrastructure, not one-off projects, is the binding requirement (Kilgus et al., 2024; Blidaru et al., 2025; Niemeyer et al., 2026).

### 9.4 Governance: from compliance to legitimacy

The success of EHDS-biobank integration depends on governance that is trusted, comprehensible and enforceable (Donia and Marelli, 2025). Compliance with legal requirements is necessary but insufficient. Participants and the public need to see that the system is fair, transparent and beneficial.

A robust governance model should include several components. First, HDABs, ethics committees and biobank access committees should have clearly differentiated roles to avoid duplication. Second, approvals should be publicly registered, with project summaries, data categories, commercial involvement and expected public benefit. Third, output checking and audit logs should be standard. Fourth, sanctions for misuse should be credible. Fifth, participants and patient representatives should have roles in governance, especially for sensitive domains such as genomics, rare diseases and commercial partnerships.

Benefit-sharing requires more explicit attention (Dove, 2024). Public discomfort often arises not from research use itself but from opaque commercial use. Industry involvement is not inherently unethical. Pharmaceutical, diagnostics and biotechnology companies are essential for clinical translation. However, commercial access to publicly contributed data and samples should be justified by transparent public benefit. This may include access fees reinvested in biobanks, return of aggregate results, diagnostic capacity-building, affordable access commitments, or royalty-in-kind mechanisms.

Governance should also distinguish between population-level and individual-level risks. Genomic research can stigmatise groups, reveal ancestry information, or affect relatives (de Vries et al., 2012; Erlich and Narayanan, 2014; Cohen et al., 2026). An individual consent model cannot fully address these risks. Community engagement, culturally sensitive governance and careful communication of population findings are needed. The distribution of implementation pressures is not uniform across the ecosystem.

Stakeholder-based analyses indicate how legitimacy can be designed rather than assumed. Studies of public and stakeholder views on justice and benefit-sharing in health-data governance (Tully et al., 2018; Bruthans et al., 2026; Laes et al., 2026), together with work on the intellectual-property and value-distribution arrangements that determine who gains from reuse (Rak, 2026) point towards transparent, negotiated benefit-sharing as a precondition for trust.

## 10 Strategic priorities for European implementation

**FIGURE 2:**
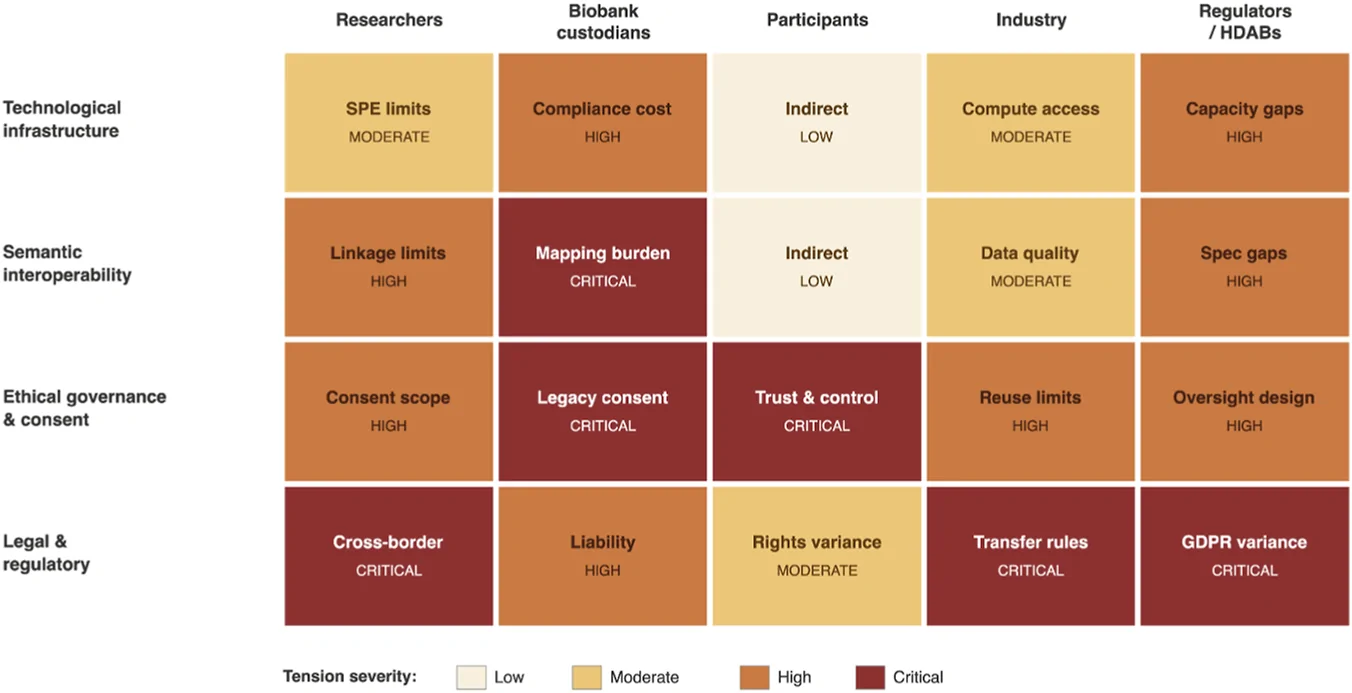
Distribution of EHDS-biobank integration tensions across challenge domains and stakeholder groups. The matrix maps the relative concentration of implementation tensions across four challenge domains and five stakeholder groups involved in EHDS-biobank integration. Challenge domains include technological infrastructure, semantic interoperability, ethical governance and consent, and legal or regulatory complexity. Stakeholders include researchers, biobank custodians, participants, industry, and regulators or Health Data Access Bodies. Colour intensity denotes the authors’ qualitative assessment of tension severity, ranging from low to critical. The figure illustrates that the most acute pressures are not evenly distributed. Biobank custodians face critical burdens around semantic mapping and legacy consent; participants face critical concerns around trust and control; researchers face critical barriers in cross-border legal implementation; industry faces critical constraints around transfer rules; and regulators or HDABs face critical pressure from GDPR variance and capacity limitations. The matrix is intended as a heuristic synthesis of the review’s argument, not as a quantitative risk score.

Four priorities should guide implementation. They respond directly to the way integration tensions are distributed across challenge domains and stakeholder groups. As summarised in Figure 2, the most acute tensions cluster around legacy consent, semantic mapping, cross-border legal uncertainty, data-transfer restrictions and HDAB capacity.

First, Europe needs harmonised interpretation of the interaction between the EHDS, GDPR, national genomic laws and biobank consent (Marelli et al., 2023; Quinn et al., 2024). Joint guidance from the EHDS Board, European Data Protection Board, BBMRI-ERIC and relevant ethics bodies should address consent-permit alignment, legacy collections, opt-out implementation, international transfer, commercial access and return of findings.

Second, infrastructure investment must be redistributive. Shared SPE hubs, common workflow libraries, reference implementations, cybersecurity support and training should be targeted towards less-resourced Member States and smaller biobanks (Svingel et al., 2025). Participation should not depend on national wealth or institutional scale.

Third, interoperability must be biobank-grade. EHDS implementing acts should incorporate biospecimen and molecular metadata standards, including MIABIS, SPREC, GA4GH Phenopackets, Beacon, htsget, VRS and FHIR-Genomics (Merino-Martinez et al., 2016; Rehm et al., 2021). Dataset catalogues should include quality, provenance, consent and bias metadata.

Fourth, trust architecture must be built into the system. Dynamic or meta-consent should be supported for new collections where feasible, but pragmatic pathways are needed for legacy collections (Kaye et al., 2015; Staunton et al., 2024). Public registers of approved projects, benefit-sharing disclosures, participant representation and accessible opt-out mechanisms should be standard. AI projects should require fairness audits and external validation.

## 11 Conclusion

The EHDS, European biobanking, and the 1+MG/GDI ecosystem represent a unique opportunity to build a federated, molecularly informed, learning health research infrastructure. The opportunity is real, but it is not self-executing. The hardest problems are not merely technical. They concern legal interpretation, standards alignment, sustainable financing, participant trust, institutional accountability and equity.

Biobanks bring biological depth and long-term stewardship. The EHDS brings legal architecture, clinical breadth and structured secondary-use pathways. GDI brings genomics-specific federation. Their integration could accelerate rare disease diagnosis, pharmacogenomics, oncology precision medicine, public health surveillance and AI validation. But poor implementation could produce a system that is formally European and practically unequal, dominated by well-resourced centres and poorly trusted by citizens.

The decisive question is therefore not whether Europe can connect health data and biobanks. It can. The question is whether it can do so in a way that is scientifically valid, legally coherent, environmentally sustainable, socially legitimate and clinically useful. That will require coordinated action during the current implementation window, before institutional habits, technical shortcuts and unequal capacity become embedded.
