# Supplementary material for: The European Health Data Space and biobanking in Europe: synergies, tensions and the future governance of data-driven health research
Source: Front Genet. 2026 Sep 10;17:1932078. doi: 10.3389/fgene.2026.1932078 (PMC13600784; doi:10.3389/fgene.2026.1932078)
Supplement: Supplementary file 1 [file Table1.docx]

**The European Health Data Space and Biobanking in Europe: Synergies, Tensions and the Future Governance of Data-Driven Health Research**

**Supplementary File**

1.0 **Literature Search Strategy**

**Database:** PubMed/MEDLINE (National Library of Medicine)

**Coverage:** 1 January 2014 - 28 July 2026

**Language:** English (applied at screening)

**Interface:** PubMed E-utilities / advanced search builder

**Table S1:** Search concept blocks

| **Block** | **Concept** | **Search terms (combined with OR)** |
| --- | --- | --- |
| 1 | European Health Data Space / secondary use | "European Health Data Space"[tiab] OR EHDS[tiab] OR "health data space"[tiab] OR "secondary use of health data"[tiab] |
| 2 | Biobanking, genomic infrastructure, federation | biobank*[tiab] OR "biological specimen banks"[MeSH] OR BBMRI[tiab] OR "1+ Million Genomes"[tiab] OR "genomic data infrastructure"[tiab] OR "genome phenome"[tiab] OR federated[tiab] |
| 3 | Governance, interoperability, standards | governance[tiab] OR interoperab*[tiab] OR consent[tiab] OR GDPR[tiab] OR "secure processing environment"[tiab] OR "data sharing"[tiab] OR standard*[tiab] |

The three blocks were combined as **Block 1 AND Block 2 AND Block 3**.

**Table S2:** Supplementary Sources

| **Source** | **Purpose** |
| --- | --- |
| EUR-Lex (eur-lex.europa.eu) | Primary EU legal instruments: GDPR (Reg. (EU) 2016/679), EHDS (Reg. (EU) 2025/327), AI Act (Reg. (EU) 2024/1689), NIS2 Directive |
| BBMRI-ERIC documentation | Biobank standards (MIABIS, SPREC), Directory/Locator/Negotiator services |
| GA4GH documentation | Genomic standards: Beacon, htsget, Phenopackets, Variation Representation Specification, Passports, Data Use Ontology |
| 1+MG / Genomic Data Infrastructure (GDI) documentation | Federated genomic infrastructure specifications |
| Backward and forward citation searching | Snowballing of reference lists and citing articles of included records |

**Inclusion and Exclusion Criteria**

Inclusion: peer-reviewed articles, authoritative policy or infrastructure reports, or primary EU legal instruments addressing one or more of:

a) the regulatory architecture or secondary-use provisions of the EHDS;
b) the organisation, interoperability, consent or governance of European biobanks;
c) genomic-data federation and standards relevant to 1+MG/GDI;
d) the legal, ethical, technical or equity dimensions of integrating these.

Exclusion:

a) not in English;
b) addressing non-European health-data systems without transferable relevance;
c) purely clinical or laboratory methods without a data-governance or infrastructure dimension;
d) duplicates.

No restriction was placed on study design given the conceptual nature of the review question

**Search results**

The PubMed/MEDLINE search identified 234 records; these were screened on title and abstract. Of these, 37 were excluded (18 addressing non-European settings without transferable relevance, 10 not in English and 9 for insufficient relevance to EHDS-biobanking integration), yielding 197 reports assessed for full-text eligibility. Following full-text assessment, 61 database-search records were incorporated into the synthesis alongside 85 sources identified through citation searching and supplementary channels (foundational literature, EU legal instruments, and standards and infrastructure documentation), giving 146 sources in total. The flow of records through identification, screening, eligibility and inclusion is shown in the PRISMA diagram below.

**PRISMA 2020 flow diagram of the identification and selection of sources. Records were identified through a structured Boolean search of PubMed/MEDLINE and supplemented by EU legal instruments, infrastructure documentation and citation searching.**

**Selection process**.

After de-duplication, two reviewers (LG, NPP) independently screened titles and abstracts against the eligibility criteria, followed by full-text assessment of potentially relevant records. Foundational and legal sources identified through supplementary and citation searching were added where they met the eligibility criteria.
